# An Exceptional Gene: Evolution of the *TSPY* Gene Family in Humans and Other Great Apes

**DOI:** 10.3390/genes2010036

**Published:** 2011-01-10

**Authors:** Yali Xue, Chris Tyler-Smith

**Affiliations:** The Wellcome Trust Sanger Institute, Wellcome Trust Genome Campus, Hinxton, Cambs. CB10 1SA, UK; E-Mail: ylx@sanger.ac.uk

**Keywords:** *TSPY*, Y chromosome, great ape, human, evolution

## Abstract

The *TSPY* gene stands out from all other human protein-coding genes because of its high copy number and tandemly-repeated organization. Here, we review its evolutionary history in great apes in order to assess whether these unusual properties are more likely to result from a relaxation of constraint or an unusual functional role. Detailed comparisons with chimpanzee are possible because a finished sequence of the chimpanzee Y chromosome is available, together with more limited data from other apes. These comparisons suggest that the human-chimpanzee ancestral Y chromosome carried a tandem array of *TSPY* genes which expanded on the human lineage while undergoing multiple duplication events followed by pseudogene formation on the chimpanzee lineage. The protein coding region is the most highly conserved of the multi-copy Y genes in human-chimpanzee comparisons, and the analysis of the d*N*/d*S* ratio indicates that *TSPY* is evolutionarily highly constrained, but may have experienced positive selection after the human-chimpanzee split. We therefore conclude that the exceptionally high copy number in humans is most likely due to a human-specific but unknown functional role, possibly involving rapid production of a large amount of *TSPY* protein at some stage during spermatogenesis.

## 1. Introduction

In 2010, we can view the *TSPY* gene family with the perspective provided by a high-quality finished sequence of the human genome [1], whole-genome sequences from multiple humans generated by the 1000 Genomes Project [2], published draft chimpanzee [3] and macaque [4] genome sequences, and unpublished draft sequences of the gorilla and orangutan which, according to general practice in genomics, are already freely available (http://www.ensembl.org/index.html). When we do this, we see that in humans *TSPY* has the unusual and striking characteristic of being carried at high copy number in a tandemly-repeated array of around 20–40 copies [5]. Most human genes are present in a single copy per haploid genome, and while duplicated genes with two copies are not unusual, protein-coding genes carried in higher copy numbers become progressively rarer [6]. No other protein-coding gene has so many copies. Why should humans have 20–40 copies of just one of their genes, *TSPY*? Many approaches to addressing this question could be taken, including studies of its biochemistry and expression or genetics, and the accompanying articles in this special issue of *Genes* explore several of these areas. Here, we take an evolutionary-genetic approach and review the history of the *TSPY* genes over the last few million years within the great apes. Have the unusual copy number and genomic organization seen in humans been a long-term characteristic of ape genomes, or are they specific to humans or a subset of apes? How rapidly has the *TSPY* amino acid sequence evolved?

We next summarize the steps that have led to our current understanding, and the resources available to address these questions. In the following sections, we present some additional evolutionary analyses based on the datasets available, and then discuss their implications for our understanding of the unusual properties of *TSPY*.

### 1.1. Human TSPY Genes

**Figure 1:**
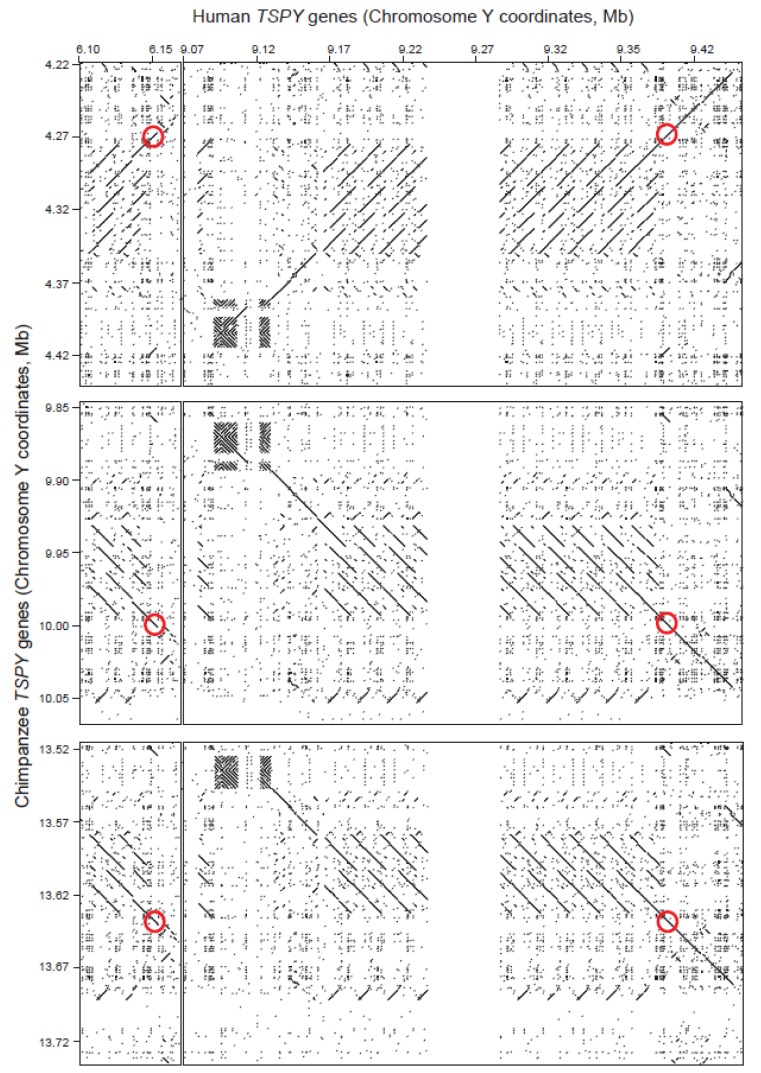
Comparison of human and chimpanzee gene clusters. Left panels, human *TSPY* minor cluster at 6.1 Mb; right panels, human *TSPY* major array at 9.1–9.4 Mb including assembly gap shown as a blank region (GRCh37). Top panels, chimpanzee *TSPY* cluster 1 at 4.3 Mb (4 genes); middle panels, chimpanzee *TSPY* cluster 2 at 9.9 Mb (1 gene); bottom panels, chimpanzee *TSPY* cluster 3 at 13.6 Mb (1 gene) (panTro2). Red circles indicate the region analyzed in Figure 2.

*TSPY* genes, as implied by their name, are located on the Y chromosome. *TSPY* was in fact one of the first human Y-chromosomal genes to be identified, in 1987 when Arnemann *et al.* published the results of a survey in which they used 18 DNA fragments enriched for Y-chromosomal sequences to search for transcripts, and found evidence for an abundant testis-specific mRNA [7]. The next year, a study of Y-chromosomal repeated sequences identified a tandemly-repeated array, *DYZ5*, on Yp, consisting of multiple homogeneous 20.3 kb units [5], and it soon became apparent that each unit carried a copy of *TSPY*. Several *TSPY*-related sequences were present elsewhere on the chromosome [8], the most substantial being designated *TSPY* minor [5]. Fuller details of the genomic structure, showing the presence of one intact *TSPY* gene at *TSPY* minor, and a more accurate unit size for the major array of 20.4 kb, were revealed by the finished sequence of the euchromatic part of the Y chromosome [9]. Nevertheless, only the edges of the array could be assembled, and a gap corresponding to most of the array remains even within the current human genome reference assembly (GRCh37/hg19; http://genome.ucsc.edu/cgi-bin/hgGateway; see Figure 1).

There is substantial variation in the number and organization of human *TSPY* genes between individuals, and this can be understood as a consequence of the complex repeated structures of the regions in which they lie. As indicated above, the number of copies in the tandem array varies, most likely as a result of homologous but unequal exchange events between sister chromatids leading to expansion and contraction of the array. Copy numbers reported in population samples range from 27–40 (*n* = 17 [5]), 18–40 (*n* = 42 [10]), 18–48 (*n* = 93 [11]) or 23–64 (*n* = 47 [12]), revealing the presence of greater than three-fold variation. In addition, a ∼4 Mb section of Yp containing the *TSPY* genes can be found in either orientation and has apparently undergone ≥12 inversion events mediated by flanking IR3 repeats during the evolutionary history of extant Y chromosomes [12], around 100,000 years. In one of these orientations, recombination can occur between DNA including the genes *AMELY, TBL1Y* and *PRKY* as well as some *TSPY* copies. Deletion carriers show no overt phenotypic effects, and the deletion is present at a frequency of ∼2% in the Indian subcontinent [14,15].

### 1.2. Ape TSPY Genes

Two factors limit comparisons between human *TSPY* genes and those of other apes. First, some reference sequences, such as that of the gorilla, have been derived from females and thus provide no information about Y-specific genes such as *TSPY.* Second, although early studies had revealed the likely presence of multiple Y-specific *TSPY* genes in other apes [7,16,17], the complexity of the *TSPY* genomic structure meant that finished sequence was necessary for detailed comparison. Fortunately, a finished Y sequence is available for the chimpanzee [18]. The title of the paper presenting this work was “*Chimpanzee and human Y chromosomes are remarkably divergent in structure and gene content*”. The authors indeed documented 41 differences in gene content between the two Y chromosomes, far in excess of findings on other chromosomes. An earlier genomewide comparison had, for example, identified just 134 gene increase and six decrease events on the human lineage [19]. But even more remarkably, 70% of the Y-chromosomal gene copy differences (involving 29 gene copies) were due to different numbers of *TSPY* genes. Although *TSPY* was the most highly repeated gene on the chimpanzee Y chromosome, with six copies (equal to *RBMY*), the uniquely high copy number of *TSPY* in humans is matched only by the uniquely large differences in copy number between humans and chimpanzees. A more detailed comparison of *TSPY* organization in the two species is provided below in Section 2.

### 1.3. Why Has TSPY Gene Copy Number Changed Rapidly in the Apes?

Two broad classes of evolutionary explanation could be considered for the extensive differences in *TSPY* copy number between humans and chimpanzees. *TSPY* might be evolving neutrally in one or both species, in which case the difference between six and ∼35 copies would have no functional significance and would be a consequence of neutral genetic drift. The alternative is that *TSPY* retains an important functional role in both species, and the different copy numbers have biological significance. The conservation of multiple *TSPY* copies on both Y chromosomes provides some evidence for functional relevance [20], and studies in humans tend to support the hypothesis of a functional role. *TSPY* is expressed specifically in spermatogonia in the testis [21], and decreased copy number has been associated with impaired spermatogenesis [22], although not in all studies [23,24]. Evolutionary analyses can also address the issue of the likely functional importance of the *TSPY* genes, and are considered below in Section 2.

## 2. Comparison of Human and Chimpanzee *TSPY* Gene Organization

### 2.1. TSPY Gene and Pseudogene Copy Numbers

Human *TSPY* genes, as noted above, are located in two regions of the Y: a large tandem array of ∼35 *TSPY* copies, and a single separate gene. Five unprocessed pseudogenes are also annotated in the reference sequence ([9], Table 1). The chimpanzee Y reference sequence, in contrast, has six active genes divided among three clusters containing four, one and one respectively, but 21 annotated unprocessed pseudogenes ([18], Table 1). Thus while the numbers of intact genes differ six-fold, the total numbers of genes plus pseudogenes are more similar, particularly when the variation within humans is taken into account.

**Table 1.** Numbers of *TSPY* gene and pseudogene copies in the human and chimpanzee reference sequences.

	***TSPY* Genes**	***TSPY* Pseudogenes**	**Total**	**Reference**
**Human**	∼35 + 1	5	∼41	[9]
**Chimpanzee**	4 + 1 + 1	21	27	[18]

### 2.2. TSPY Cluster Relationships

In order to investigate the relationships between the two human and three chimpanzee *TSPY* clusters, we compared them and their flanking sequences using the program DOTTER [25]. Single-copy sequences with high similarity produce a diagonal line in such an analysis; tandem repeats produce a series of diagonal lines offset by the size of the repeating unit. The results of this analysis are shown in Figure 1. We can draw four main conclusions. First, the three panels representing the three chimpanzee *TSPY* clusters are broadly similar, apart from the different orientation of the cluster at 4.3 Mb. Second, the similarity between all of the different gene copies in the two species extends far outside the coding region to the entire repeat unit. This is expected from the locations of each chimpanzee *TSPY* cluster within one of the “pink” amplicons [18]. Third, the chimpanzee *TSPY* genes are organized into tandem arrays, but these are much smaller than the major human array, consisting of around four repeat units, and all have substantial deletions disrupting their regular structures. This is consistent with the inactive nature of the majority of the chimpanzee *TSPY* copies. Fourth, there is similarity between the sequences flanking the human major array on both sides and the sequences flanking the chimpanzee arrays. The sequences flanking the minor human array are less closely related.

**Figure 2:**
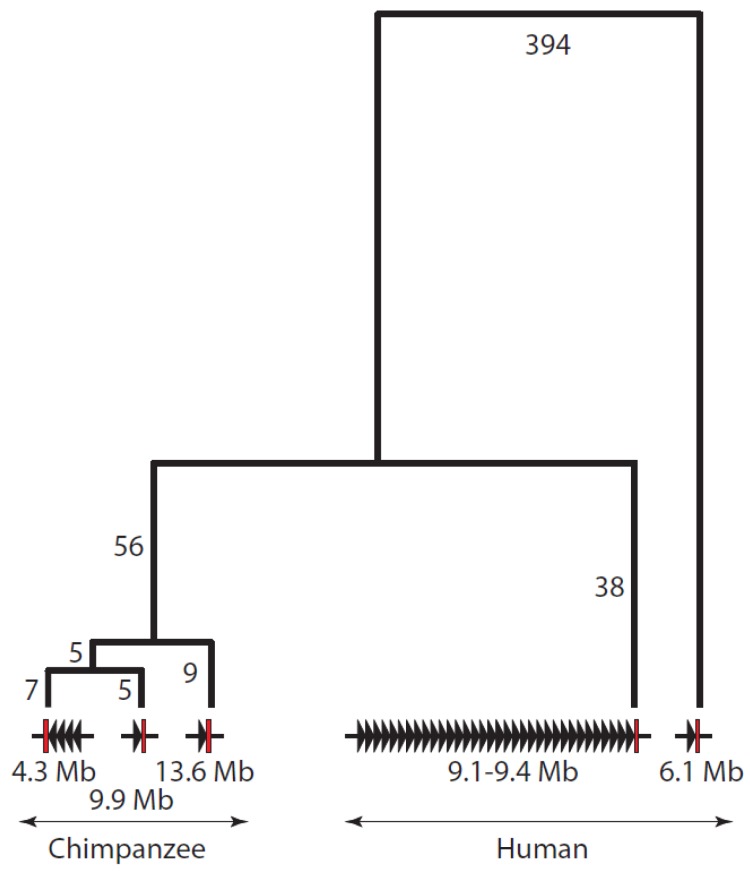
Phylogenetic relationships of the two human and three chimpanzee *TSPY* arrays deduced from comparisons of the flanking sequences circled in Figure 1 (coordinates in text). The arrays, named according to their approximate genomic coordinates, are shown at the bottom, with active *TSPY* genes indicated by black arrowheads and the flanking region by a red rectangle. The topology of the tree is shown and the number of mutational events on each branch indicated, but the branches are not drawn to scale. Note that no outgroup sequence is available and the tree is unrooted.

To examine the phylogenetic relationships between the five *TSPY* clusters in more detail, we identified a flanking region with high sequence similarity (within the red circles in Figure 1), aligned the five sequences using ClustalW2 (http://www.ebi.ac.uk/Tools/msa/clustalw2/) [26] and corrected the alignment manually. The alignment spanned 6277 bp and came from two nearby segments (human 6,147,115–6,148,562 and 6,150,328–6,155,088; 9,400,312–9,401,761 and 9,403,547–9,408,316; chimpanzee 4,275,601–4,277,071 and 4,269,058–4,273,824; 9,990,626–9,992,099 and 9,993,868–9,998,636; 13,631,075–13,632,544 and 13,634,296–13,639,063). After excluding length variations in mononucleotide runs, variable positions consisting of base substitutions or insertions/deletions were identified and, if immediately adjacent, assigned to a single mutational event. In this way, 515 mutational events were identified, 514 of which were consistent with a simple parsimony tree. This unrooted tree is shown in Figure 2.

From these two analyses, we can see that the human minor array is distinct from all the other arrays because most of its flanking regions do not align with them (Figure 1), and even where there is good alignment, it is the most divergent sequence (Figure 2). In contrast, the human major array and its flanking sequences show strong sequence similarity to the three chimpanzee arrays (Figure 1). The phylogenetic reconstruction, which in this part of the tree can be rooted using the human minor array as an outgroup, shows that the chimpanzee arrays were all derived from a shared common ancestor on the chimpanzee lineage after the human-chimpanzee split, and that the arrays at 4.3 and 9.9 Mb are the most closely related. Since homologous clusters of multiple *TSPY* genes are found in both species, the common ancestor is likely to have carried such a cluster, and if this is the case, the multiple *TSPY* pseudogenes in the chimpanzee arrays are likely to have been generated independently from functional genes on the chimpanzee lineage. Since the large number of structural differences between the human and chimpanzee Y chromosomes, and the extensive structural polymorphism even within humans, suggest that large numbers of rearrangements have occurred during the descent from this ancestral structure, the intermediate steps cannot be reconstructed in more detail using the current data. The lack of an outgroup, such as a finished gorilla Y chromosome sequence, also limits the deductions we can make about the ancestral structure, although cytogenetic analyses show one signal of intermediate intensity in gorillas [16]. Nevertheless, a simple model consistent with the data available would propose a moderate-sized ancestral tandem array of *TSPY* genes in the human-chimpanzee ancestor, with expansion on the human lineage contrasted with array duplications and pseudogenization on the chimpanzee lineage.

## 3. Evolution of the *TSPY* Amino acid Sequence

### 3.1. Divergence of the Coding Sequence between Humans and Chimpanzees

Here we return to the question of whether the rapid evolution of the *TSPY* genes is more likely to represent relaxation of constraint or selection for different functional configurations in humans and chimpanzees. If the *TSPY* genes were functionally unimportant in either species, we would expect to see an increase in the rate of nucleotide substitutions in the coding region, which might approach the rate in predominantly neutral regions of the genome such as introns. Hughes and colleagues [18] tabulated a comparison of the extent of divergence of the ampliconic gene sequences, divided into coding and intronic sequences (Table 2).

**Table 2.** Divergence of human and chimpanzee ampliconic gene sequences. All data are from [18]; NA = not applicable.

	**% Coding Divergence**	**% Intron Divergence**
***BPY2***	0.935	1.504
***CDY***	1.705	NA
***DAZ***	2.188	1.422
***RBMY***	2.728	2.511
***TSPY***	0.794	2.888
***VCY***	4.298	1.579
***XKRY***	1.626	NA

Table 2 shows that introns have diverged by 1.4 to 2.9%, similar to the overall average figure 1.7% for the X-degenerate regions of the Y chromosomes. In contrast, the *TSPY* coding region shows 0.8% divergence, substantially lower. These conclusions are consistent with an earlier study that examined part of exon 1, exon 2 and intron 1, and estimated 1.9% divergence for the intron-exon segment as a whole [27]. *TSPY* shows the lowest coding divergence of any of the ampliconic genes, but the highest intronic divergence. It therefore seems unlikely that ampliconic organization or a reduced mutation rate could account for the low coding divergence: functional constraint provides the best explanation.

### 3.2. The Ratio of Synonymous and Non-Synonymous Changes

To investigate this coding divergence in more detail, we compared the rates of synonymous substitutions, which do not change the coding sequence and are usually neutral, with those of non-synonymous, which alter the coding sequence, using the d*N*/d*S* statistic implemented in DNaSP (http://www.ub.edu/dnasp/). For these comparisons, we chose the two alternative CCDS annotations of *TSPY* as the human sequences (CCDS48204, CCDS48205), the chimpanzee sequence (ENSPTRT00000055849 with manual annotation according to information from the Page lab and new alignment id), and used the *TSPY* sequence from a new world monkey, the marmoset, as an outgroup (ENSCJAG00000034791). The results are shown in Table 3.

**Table 3.** Selective forces on the *TSPY* amino acid sequence assessed using the d*N*/d*S* statistic.

**Comparison**	**Non-Synonymous Differences**	**Synonymous Differences**	**d*N*/d*S***
**CCDS 48204**			
**Marmoset-human**	71	35	0.58
**Marmoset-chimpanzee**	73	34	0.61
**Human-chimpanzee**	5	1	1.49
**CCDS 48205**			
**Marmoset-human**	73	34	0.62
**Marmoset-chimpanzee**	73	35	0.61
**Human-chimpanzee**	7	0	NA

A d*N*/d*S* value below 1 indicates purifying selection, a value of 1 neutrality, and a value above 1 positive selection. The marmoset provides a relatively distant outgroup, and this has the advantage that many differences between the marmoset-great ape sequences have accumulated. The d*N*/d*S* values between marmoset and either human or chimpanzee are similar, and less than 1, showing that selection has acted to reduce the number of amino acid changes: purifying selection is seen here, as is expected to predominate in protein-coding regions. This supports the idea that these *TSPY* sequences are functional, and have been for most of their evolutionary history. The human-chimpanzee value, in contrast, is positive for CCDS 48204 and undetermined for CCDS 48205 since there are no synonymous differences in this comparison. If the number of synonymous differences were conservatively set to 1 instead of zero, the d*N*/d*S* value would be 2.11. These positive values could indicate positive selection for change in the amino acid sequence of *TSPY* since the human-chimpanzee split. However, this interpretation needs to be considered cautiously: if there had been two synonymous differences in each case, d*N*/d*S* would be close to 1, consistent with neutrality. However, the low number of human-chimpanzee differences in the *TSPY* coding region, compared with introns and other genes (Table 2) is not consistent with neutrality. In all, the analyses of the *TSPY* coding region support the idea of purifying selection on the *TSPY* sequence for most of its history, and reveal likely positive selection in the last few million years.

## 4. Conclusions

We wished to understand why humans have so many copies of one of their genes, *TSPY*. Within mammals, *TSPY* copy number is quite variable: mice, for example, have only an inactive copy [28,29], although rats appear to have one functional copy [28], and the copy number within cattle has been reported to vary between about 50 and 200 copies [30,31], although it remains unclear how many of these copies are functional. Within the great apes, *TSPY* copies appear from *in situ* hybridization studies to metaphase chromosomes (which do not distinguish between genes and pseudogenes) to be present at moderate levels in gorillas and bonobos, and at higher levels in orangutans, chimpanzees and humans [16]. *TSPY* is clearly not essential in all mammals, and varies substantially in copy number between even closely related species. Yet the conservation of its protein sequence, both as recognizable homologs in many mammals, and more specifically within the great apes as shown in Section 3 above, points to a functional role. *TSPY* is a member of the broad NAP/SET protein family [32], so a function as a protein chaperone might be sought.

Expression is predominantly in the tests, in spermatogonia, where the protein is seen mainly in the cytoplasm, and varies in extent between cells, being highest in adjacent pairs of cells involved in spermatogonial proliferation, together with a little staining in spermatocytes [21]. This suggests that its role may lie in male reproduction. It is notable that genes involved in reproductive processes are often found to evolve rapidly, showing signals of positive selection in genome-wide surveys. For example, the GO category “gametogenesis” showed evidence for unusually high levels of positive selection in a survey of human protein-coding genes [33], and the categories “gametogenesis”, “spermatogenesis and motility” and “fertilization” all showed similar evidence in a survey detecting positive selection in the last ∼30,000 years [34]. In addition, other differences between humans and chimpanzees in Y-encoded genes implicated in spermatogenesis have been identified [35]: inactivation of USP9Y in humans is usually [36] but not invariably [37] associated with spermatogenic failure, while chimpanzees and bonobos carry an inactive copy inherited from their common ancestor [38] who may have lived more than one million years ago, without manifesting defects in spermatogenesis. *TSPY* may therefore be a member of a class of genes involved in spermatogenesis that continue to evolve rapidly in a variety of ways.

We conclude that *TSPY* is likely to be present at such high copy number in humans because multiple copies have conferred a selective advantage on the human lineage. Although high copy numbers are unusual for protein-coding genes, RNA-coding genes are often highly repeated [39]. Here, no increase in product level by regulation of translation or protein turnover is possible, so more genes are needed to produce more product. Among protein-coding genes, a rare informative example of high copy number is provided by the sea urchin histone genes [40]. Histones are the major structural proteins in chromatin, and high levels are needed for rapid cell division during early development. These examples, together with the expression data, suggest that large amounts of *TSPY* protein are advantageous at some stage during spermatogenesis, and that this advantage is particularly marked in humans. Investigation of male Neanderthal or Denisovan genomes might provide some information on when the *TSPY* copy number increased and how specific this increase was to modern humans. Thus an evolutionary consideration of the *TSPY* gene family suggests multiple further directions for future research.
